# Minority hair tax: pricing bias in haircare products

**DOI:** 10.1097/JW9.0000000000000089

**Published:** 2023-06-16

**Authors:** Yacine N. Sow, Amanda A. Onalaja-Underwood, Tiaranesha K. Jackson, Susan C. Taylor, Temitayo A. Ogunleye

**Affiliations:** a Morehouse School of Medicine, Atlanta, Georgia; b Department of Dermatology, Vanderbilt University Medical Center, Nashville, Tennessee; c University of Illinois Chicago College of Medicine at Peoria, Peoria, Illinois; d Department of Dermatology, Perelman School of Medicine at the University of Pennsylvania, Pennsylvania, Philadelphia

**Keywords:** ethnic tax, hair typing, haircare, natural hair, price discrimination

## Abstract

**Background:**

Black haircare is an estimated $2.51-billion-dollar industry. Black women spend 9 times more on ethnic hair products than non-Black consumers. The haircare industry has adapted to these market trends by developing products catering to the needs of “natural hair,” referring to curly to tightly coiled hair texture that has not been chemically straightened with a relaxer. Anecdotally, natural haircare products are relatively expensive.

**Objective:**

We aimed to investigate texture-based price differences for haircare products targeting coily/curly compared to straight hair types.

**Methods:**

Data were collected in August 2022 from 6 brands available on www.amazon.com. After stratifying the data by manufacturer, hair texture, and average price/oz, we used 2 sample *t*-test with equal variances to examine cost differences.

**Results:**

Overall, there was a significant difference in average price/oz between all coily/curly and straight hair products, with coily/curly hair products being more expensive. When stratified by manufacturer, one leading US manufacturer sold coily/curly hair products at a higher price ($0.66/oz ±$0.05) compared with straight hair products ($0.46/oz ±$0.04), *t*_14_ = 2.8967, *P* < 0.0134.

**Limitations:**

A small sample size of only shampoos and conditioners were analyzed, which may not represent the number of haircare products that consumers use.

**Conclusion:**

Pricing policies should ensure that all individuals have access to effective, affordable haircare products. Dermatologists should also be cognizant of pricing differences to direct patients with natural hair to fairly priced products.

**What is known about this subject in regard to women and their families?**

- Natural hair refers to a curly to tightly coiled hair texture that has not been chemically straightened.
- Considering that natural hair is structurally fragile, tailored haircare products are formulated to reduce breakage and assist with styling.
- Anecdotally, haircare products tailored for natural, coily, curly, and kinky hair types are relatively more expensive than products tailored for straight hair types.

**What is new from this article as messages for women and families?**

- We evaluated if there were texture-based price differences for haircare products targeting coily/curly compared to straight hair types.
- Coily/curly haircare products were significantly more expensive in average price/oz compared to haircare products for straight hair types.
- This study provides further evidence of texture-based price discrimination in haircare products that should be addressed through policy change.

## Introduction

“Natural” refers to the texture of curly to tightly coiled hair that has not been chemically straightened with a relaxer. Hair of African ancestry has curved follicles, an elliptical shape on cross-section, and numerous twists in hair strands, leading to an increased susceptibility to hair breakage.^1^ Thus, natural hair may require products specifically formulated to reduce breakage. Dermatologists often support natural hairstyling, particularly for patients with conditions such as traction alopecia and central centrifugal cicatricial alopecia. Anecdotally, natural haircare products are relatively expensive. Consumers of color make significant contributions to the overall haircare industry as African Americans contributed $54 million of the $63 million spent in the ethnic hair and beauty market in 2017.^2^ The buying potential of Black consumers has driven haircare brands to produce more products targeting this group. Price controls and restrictions are limited in the United States, which may lead to consumer price discrimination. For instance, a recent study noted that over-the-counter women’s 5% minoxidil had a 40% higher average price than men’s 5% minoxidil.^3^ To our knowledge, there is no published research on texture-based price discrimination. Thus, we sought to investigate differences in pricing for haircare products targeting coily/curly versus straight hair types.

## Materials and Methods

Data were collected in August 2022 on Amazon.com from 6 brands that produce separate haircare products for both straight and coily/curly hair types. A product was considered to target natural hair if the adjectives “natural,” “curly,” “coily,” or “kinky” were on the product or if it was in the “curly” or “textured” hair type category. Shampoos and conditioners were examined. Gels, foams, leave-in conditioners, hairsprays, deep conditioners, and 2 in 1 shampoo and conditioner were excluded due to a lack of comparable products for both hair types. Additionally, individual haircare products advertised for all hair types, formulated for processed or colored treated hair, containing minoxidil or DHT blocker, and/or sold by Amazon resellers were all excluded. The “subscribe and save” option on Amazon was not selected when investigating prices. Products were stratified by manufacturer, targeted hair texture (coily/curly versus straight), average price per ounce, and compared using 2-sample *t* tests with equal variances. Two-sided *p* values <.05 were considered significant. Analysis was performed using STATA, version 17 (StataCorp LLC). This study was Institutional Review Board exempt.

## Results

A total of 48 haircare products (24 shampoos and 24 conditioners) were included, and 6 brands met the inclusion criteria: Head & Shoulders, L’Oréal Paris, Pantene, Dove, TRESemmé, and Garnier Fructis (Table 1). There was a significant difference in average price/oz between all coily/curly ($0.56 ±$0.053) and straight hair products ($0.39 ±$0.03), *t*(46) = 3.0392, *p* < .0039, with coily/curly hair products being $0.17 per ounce more expensive. When stratified by manufacturer, Procter & Gamble (Head & Shoulders and Pantene) had a significant difference in average price/oz with coily/curly hair products ($0.66/oz ±$0.05) being more expensive compared with straight hair products ($0.46/oz ±$0.04), *t*(12) = 2.8967, *p* < .0134. Although the other manufacturers had a higher average price/oz for coily/curly products, only Procter & Gamble had a statistically significant difference (Table 2).

**Table 1:** Average price/oz of natural and straight hair products

				Number of products averaged	
	Product	Coily/curly average/oz	Straight hair average/oz	Coily/curly hair	Straight hair
Shampoos	Head & Shoulders	0.49	0.39	1	2
	L’Oréal Paris	0.635	0.27	4	2
	Pantene	0.76	0.555	3	2
	Dove	0.55	0.325	1	2
	TRESemmé	0.67	0.50	1	2
	Garnier Fructis	0.23	0.37	1	3
Conditioners	Head & Shoulders	0.49	0.62	1	1
	L’Oréal Paris	0.61	0.3075	2	4
	Pantene	0.67	0.35	2	2
	Dove	0.61	0.56	1	3
	TRESemmé	0.305	0.34	2	3
	Garnier Fructis	0.23	0.22	1	2

**Table 2:** Average price/oz of natural and straight hair products by manufacturer

	Average price/oz ($)	Standard error	Difference in means ($)	*P* value	*t* statistic	Degrees of freedom
Overall						
Coily/curly (N = 20)	0.56	0.053	0.18	0.0039^a^	3.0392	46
Straight (N = 28)	0.39	0.030				
L’Oréal USA, Inc						
Coily/curly (N = 8)	0.52	0.12	0.22	0.0609	2.0076	17
Straight (N = 11)	0.30	.05				
Procter & Gamble						
Coily/curly (N = 7)	0.66	0.05	0.20	0.0134^a^	2.8967	12
Straight (N = 7)	0.46	0.04				
Unilever						
Coily/curly (N = 5)	0.48	0.08	0.05	0.5626	0.5942	13
Straight (N = 10)	0.43	0.05				

L’Oréal USA, Inc: Garnier and L’Oréal Paris. Procter & Gamble: Head & Shoulders and Pantene. Unilever: Dove and TRESemmé.

a Two-sided *P* values <.05 were considered significant.

## Discussion

This study demonstrates an overall pricing bias of shampoos and conditioners for coily/curly and straight hair but has limitations. Most sampled products were from brands that traditionally market for straight hair types. Although these brands have developed product lines specifically catering to ethnic haircare, consumers with coily/curled textured hair may not seek or select the products we sampled. Additionally, our analysis had a small sample size of only shampoos and conditioners. This is not fully representative of the number of haircare products that consumers with coily/curly hair use, considering that oils, gels, leave-in conditioners, deep conditioners, and other stylers may be a part of styling and maintenance for natural hair. Future research should evaluate cost differences across more brands and additional product types.

Pricing of haircare products varied by brand and manufacturer. Although many brands have similar pricing per ounce, L’Oréal Paris shampoos and conditioners and Pantene conditioners charged nearly double per ounce for products targeting coily/curly hair (Table 1). This could be due to variations in ingredients or formulations. For instance, L’Oréal Paris and Procter and Gamble shampoos and conditioners had several ingredients that differed between coily/curly and straight hair products. Their coily/curly hair shampoos and conditioners had more emollients, while the L’Oréal Paris shampoo for straight hair types contained a higher number of perfume (Table 3). Overall, the shampoo for coily/curly hair types had a higher number of ingredients listed on the products compared to shampoo for straight hair types. Furthermore, many natural hair products are new additions (eg, Head and Shoulders Royal Oils Collection, Dove Amplified Textures) to the manufacturer’s product lines, and manufacturers may increase prices to compensate for research and development.

**Table 3:** Ingredient types in haircare products marketed for coily/curly and straight hair types

Ingredient type	Shampoo		Conditioner	
	Product A	Product B	Product C	Product D
	Straight	Coily/curly	Straight	Coily/curly
First 5 ingredients	-Aqua/water/eau -Sodium laureth sulfate -Sodium lauryl sulfate -Cocamide MEA -Glycol distearate	-Aqua/water -Sodium laureth sulfate -Coco-betaine -Ci 19140/Yellow 5 -Chamomilla recutita flower extract/Matricaria flower extract	-Pyrithione zinc (0.5 %) -Water -Stearyl alcohol -Cetyl alcohol -Stearamidopropyl dimethylamine	-Pyrithione zinc (0.5 %) -Water -Stearyl alcohol -Cetyl alcohol -Stearamidopropyl dimethylamine
Emollient				
Amodimethicone		X		
Caprylic/capric glycerides		X		
Caprylic/capric triglyceride		X		
Cetyl alcohol			X	X
Coconut oil		X		X
Dimethicone	X		X	X
Glycol distearate	X			
Sunflower seed oil		X		
PPG-5-Ceteth-20	X			
Stearyl alcohol			X	X
Total	3	5	3	4
Fragrance/perfume				
Alpha-isomethyl ionone	X	X		
Amyl cinnamal	X			
Benzyl alcohol	X	X	X	X
Benzyl Salicylate	X			
Citronellol	X	X		
Coumarin	X			
Limonene	X	X		
Linalool	X	X		
Parfum/fragrance	X	X	X	X
Total	9	6	2	2
Moisturizer/humectant				
Glutamic acid	X		X	
Propylene gylcol		X		
Total	1	1	1	0
All ingredients	30	35	14	12

Product A: L’Oréal Elvive Total Repair 5 Repairing Shampoo. Product B: L’Oréal Elvive Extraordinary Oil Curl Nourishing Shampoo. Product C: Head & Shoulders Smooth and Silky Dandruff Conditioner. Product D: Head and Shoulders Conditioner, Royal Oils Collection with Coconut Oil for Natural and Curly Hair.

Nevertheless, the results of our analysis posit that there may be an element of texture-based price discrimination. The natural hair industry is lucrative, in high demand, and has a large segment of Black and Latinx consumers driving haircare sales. Brands that traditionally provided straight hair products have responded to these market trends by curating product lines specifically targeting natural, coily/curly textures. The concept of an “ethnic tax” is documented with cases of salons that charge clients with natural hair more than clients with straight hair for the same service.^4,5^ Although several policies prohibit gender-based pricing discrimination in retail and service settings, “ethnic taxing” has yet to be similarly addressed in local or state laws.^6–8^ As the ethnic haircare industry grows, local policies should ensure that all people, regardless of hair texture, have access to effective and affordable haircare products. Dermatologists should also be cognizant of these price differences to direct patients to fairly priced products.

## Conflicts of interest

None.

## Funding

None.

## Study approval

N/A.

## Author contributions

YNS participated in performance of the research, data analysis, and writing of the paper. AAO participated in research design, performance of the research, and data analysis. TKJ participated in writing of the paper. SCT participated in the writing of the paper. TAO participated in research design, data analysis, and writing of the paper.

## References

[1] Aguh C Okoye GA, editors. Fundamentals of ethnic hair: the dermatologist’s perspective. 1st ed. Cham: Springer International Publishing: Imprint: Springer; 2017. doi:10.1007/978-3-319-45695-9.

[2] Black Impact: consumer categories where African Americans move markets. Published February 2018. [cited 2022 September 15]. Available from: https://www.nielsen.com/insights/2018/black-impact-consumer-categories-where-african-americans-move-markets/.

[3] Wehner MR Nead KT Lipoff JB. Association between gender and drug cost for over-the-counter Minoxidil. JAMA Dermatol 2017;153:825–6. 28593214 10.1001/jamadermatol.2017.1394 PMC5817599

[4] Store accused of charging more for “black hair.” NBC News. Published May 31, 2005. [cited 2022 September 15]. Available from: https://www.nbcnews.com/id/wbna8049156#.XFsX5FVKiUk.

[5] Rosenstien J. Why are salons charging extra to cut and style ethnic hair? Allure. Published November 2014. [cited 2022 September 15]. Available from: https://www.mmsmith.co/healthhaircare/2017/5/22/why-are-salons-charging-extra-to-cut-and-style-ethnic-hair.

[6] Rank JE. Is Ladies’ night really sex discrimination: public accommodation laws, De minimis exceptions, and stigmatic injury. Seton Hall L Rev. 2005;36:223.

[7] Abdou DS. Gender-based price discrimination: the cost of being a woman. PBES 2019;2(5):32–8. doi:10.26689/pbes.v2i5.729.

[8] Trégouët T. Gender-based price discrimination in matching markets. Int J Ind Organ 2015;42:34–5.

